# Regorafenib plus toripalimab in patients with metastatic colorectal cancer: a phase Ib/II clinical trial and gut microbiome analysis

**DOI:** 10.1016/j.xcrm.2021.100383

**Published:** 2021-08-27

**Authors:** Feng Wang, Ming-Ming He, Yi-Chen Yao, Xia Zhao, Zhi-Qiang Wang, Ying Jin, Hui-Yan Luo, Ji-Bin Li, Feng-Hua Wang, Miao-Zhen Qiu, Zhi-Da Lv, De-Shen Wang, Yu-Hong Li, Dong-Sheng Zhang, Rui-Hua Xu

**Affiliations:** 1 Department of Medical Oncology, Sun Yat-sen University Cancer Center, State Key Laboratory of Oncology in South China, Collaborative Innovation Center for Cancer Medicine, Guangzhou 510060, China; 2 Cancer microbiome platform, Sun Yat-sen University Cancer Center, State Key Laboratory of Oncology in South China, Collaborative Innovation Center for Cancer Medicine, Guangzhou 510060, China; 3 Department of Microbiology, Army Medical University, Chongqing 400038, China; 4 Department of Clinical Research, Sun Yat-sen University Cancer Center, State Key Laboratory of Oncology in South China, Collaborative Innovation Center for Cancer Medicine, Guangzhou 510060, China; 5 Research Unit of Precision Diagnosis and Treatment for Gastrointestinal Cancer, Chinese Academy of Medical Sciences, Guangzhou 510060, China

**Keywords:** colorectal cancer, immunotherapy, regorafenib, toripalimab, programmed cell death protein 1, microbiome

## Abstract

This is a phase Ib/II study of regorafenib plus toripalimab for colorectal cancer. The objective response rate (ORR) is 15.2% and the disease control rate is 36.4% in evaluable patients with recommended phase II dose (80 mg regorafenib plus toripalimab). The median progression-free survival (PFS) and the median overall survival are 2.1 months and 15.5 months, respectively. Patients with liver metastases have lower ORR than those without (8.7% versus 30.0%). All patients (3/3) with lung-only metastasis respond, whereas no patients (0/4) with liver-only metastasis respond. 94.9% and 38.5% of patients have grade 1 and grade 3 treatment-related adverse events, respectively. Gut microbiome analysis of the baseline fecal samples shows significantly increased relative abundance and positive detection rate of *Fusobacterium* in non-responders than responders. Patients with high-abundance *Fusobacterium* have shorter PFS than those with low abundance (median PFS = 2.0 versus 5.2 months; p = 0.002).

## Highlights

Regorafenib plus toripalimab improves response and overall survival
Patients with liver metastasis have lower response rate than those without
Increased *Fusobacterium* is found in non-responders compared with responders

## Graphical abstract

**Figure:**
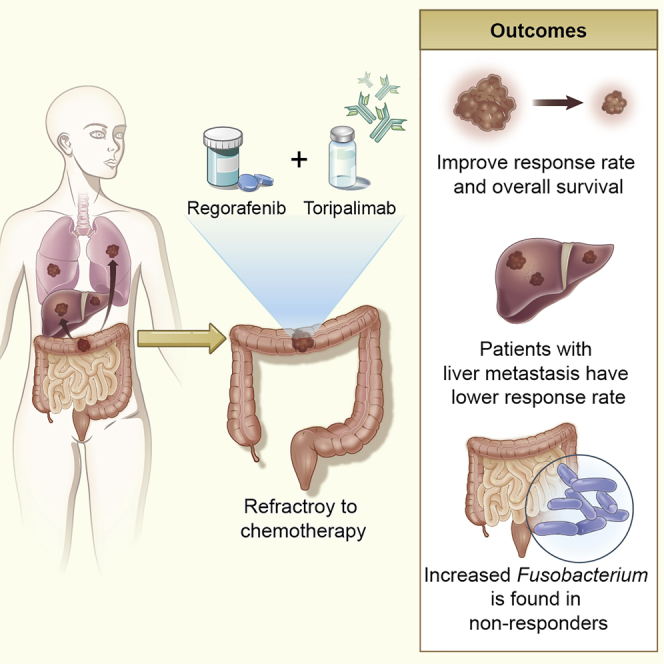

## Teaser

Wang et al. demonstrate the safety, efficacy, and survival of regorafenib plus toripalimab in colorectal cancer patients. They show the gut microbiome results that *Fusobacterium* is negatively correlated with response and survival. This provides a combination regimen for unselected refractory metastatic colorectal cancer patients.

## Introduction

Globally, colorectal cancer (CRC) is the third most common cancer and the second leading cause of cancer death.^1^ In recent years, the quality of care for metastatic CRC (mCRC) has been continuously ameliorating over time.^2^ However, for refractory mCRC, therapeutic options are still limited. Regorafenib is a multi-kinase inhibitor against vascular endothelial growth factor receptors (VEGFRs) and other kinase receptors to suppress tumor proliferation, metastasis, angiogenesis, and immune escape. As one of the standard salvage-line therapies for mCRC, its objective response rate (ORR) is only 1%–4%.^3^^,^^4^ The benefit of immune checkpoint blockade is limited to microsatellite instability-high (MSI-H) or DNA deficient mismatch repair (dMMR) mCRC and is recommended by National Comprehensive Cancer Network (NCCN) in its 3^rd^-line treatment.^5^, ^6^, ^7^ In contrast, microsatellite stable (MSS) or MMR-proficient (pMMR) mCRC with a poor immune cell infiltration,^8^ constituting ~95% of mCRC, is typically unresponsive to programmed cell death protein 1 (PD-1) blockade (ORR 0%).^5^^,^^9^ Therefore, new combination therapies are needed to improve outcomes of refractory MMS/pMMR mCRC.

Anti-angiogenic molecules, which target VEGF/VEGFR axis, can counteract the tumor-induced immunosuppression by reducing regulatory T cells and increasing CD8^+^ T cell infiltration.^10^^,^^11^ In addition, regorafenib reduced tumor-associated macrophages in tumor models by inhibiting other targets, including colony-stimulating factor 1 receptor.^12^ In murine models, the combination of regorafenib plus PD-1 blockade exhibited synergistic tumor growth suppression compared with either treatment alone.^13^ However, VEGF/VEGFR inhibition plus PD-1 blockade demonstrated inconsistent efficacy in refractory mCRC^14^, ^15^, ^16^ and failed in maintenance setting.^17^ Moreover, the combinations of regorafenib and PD-1/programmed cell death ligand 1 (PD-L1) blockade reported different outcomes. Recently, a phase Ib REGONIVO study demonstrated a high response rate of regorafenib plus nivolumab in MSS/pMMR refractory mCRC and attracted much attention in this field.^18^ However, no biomarkers were identified in this study. Another phase II REGOMUNE study of regorafenib plus avelumab, a PD-L1 monoclonal antibody (mAb), reported preliminary results of 0% ORR and grade 3 to 4 toxicity in 87% of patients with non-MSI-H refractory mCRC.^19^

Toripalimab, a recombinant, humanized immunoglobulin G4 (IgG4) monoclonal antibody against PD-1, was first approved by the National Medical Product Administration for the treatment of 2^nd^-line metastatic melanoma in China in 2018. We have demonstrated in a multi-center POLARIS-02 trial that toripalimab had an ORR of 20.5% and grade 3+ toxicity rate of 28% in patients with refractory nasopharyngeal carcinoma,^20^ which received Breakthrough Therapy designation from US Food and Drug Administration (FDA). Our previous phase-I clinical trial found toripalimab was well tolerated and demonstrated anti-tumor activity in treatment-refractory advanced solitary malignant tumors.^21^ The phase Ib/II trial also found that toripalimab monotherapy achieved similar response rate with pembrolizumab or nivolumab in unselected heavily pretreated gastric cancer patients.^22^ Recently, the combination of toripalimab and the VEGFR inhibitor axitinib showed encouraging efficacy in patients with mucosal melanoma, which otherwise had poor response to anti-PD-1 monotherapy.^23^ Accumulating evidences supported that the gut microbiome was associated with the efficacy of immune checkpoint inhibitors (ICIs) in several cancers, including non-small cell lung cancer (NSCLC), CRC, renal cell carcinoma (RCC), and melanoma.^24^, ^25^, ^26^ A recent study found that regorafenib-induced toxicity was arisen from the reactivation of the inactive regorafenib-glucuronide to regorafenib in the gastrointestinal tract by gut microbial β-glucuronidase (GUS) enzymes.^27^ However, it remains unknown whether the clinical efficacy of regorafenib or regorafenib plus ICIs was correlated with the gut microbiome.

As refractory mCRC remained an unmet medical need, we initiated the REGOTORI study in January 2019, as a two-part, dose escalation and dose expansion phase Ib/II study evaluating the tolerability, safety, preliminary efficacy, and efficacy-related gut microbiota of regorafenib plus toripalimab for patients with refractory pMMR/MSS/MSI-low (MSI-L) mCRC.

## Results

### Patient characteristics

Forty-two patients were enrolled in phase Ib/II trial between March 2019 and January 2020; 7.7% of patients had Eastern Cooperative Oncology Group (ECOG) performance scores at 0. All patients had received ≥2 previous lines of chemotherapy and were refractory to or intolerant of fluorouracil, oxaliplatin, and irinotecan. Anti-VEGF therapy (i.e., bevacizumab) and anti-epidermal growth factor receptor (EGFR) therapy (i.e., cetuximab) were used in 59.5% and 28.6% of patients, respectively. Patients were heavily pretreated with a median of 2.40 prior lines of treatments, and 69% had ≥2 metastatic sites. All patients were MSS/pMMR/MSI-L. 21 (50%) had *RAS* mutations and 2 (4.8%) harbored *BRAF*^*V600E*^ mutations (Table 1).

**Table 1:** Characteristics of patients

Characteristic	80 mg (n = 39)	120 mg (n = 3)
Age, median (range)	53 (37–69)	44 (37–55)
BMI, median (range)	22.7 (17–31.8)	26.0 (21.5–28.3)

**Gender**		

Male	20 (51.3)	2 (66.7)
Female	19 (48.7)	1 (33.3)

**ECOG performance status, n (%)**		

0	3 (7.7)	0 (0.0)
1	36 (92.3)	3 (100.0)

**Primary site**		

Right colon	13 (33.3)	1 (33.3)
Left colon/rectum	26 (66.7)	2 (66.7)

**Site of metastases**		

Liver	27 (69.2)	3 (100.0)
Lung	23 (59.0)	1 (33.3)
Lymph node	18 (46.2)	1 (33.3)
Peritoneum	10 (25.6)	0 (0.0)
Other	6 (15.4)	0 (0.0)
Chemo-refractory	32 (82.1)	3 (100.0)
Chemo-intolerant	7 (17.9)	0 (0.0)
Anti-EGFR, n (%)	10 (25.6)	2 (66.7)
Anti-VEGF, n (%)	24 (61.5)	1 (33.3)

**Prior treatment lines**		

Median, range	2 (2–5)	3 (2–3)
=2	26 (66.7)	1 (33.3)
≥3	13 (33.3)	2 (66.7)

**MSI/MMR status**		

MSS/pMMR	38 (97.4)	0 (0)
MSI-L	1 (2.6)	0 (0)

***RAS/BRAF^V600E^* status**		

*RAS* and *BRAF*^*V600E*^ wild	13 (33.3)	2 (66.7)
*RAS* mutant	20 (51.3)	1 (33.3)
*BRAF*^*V600E*^ mutant	2 (5.1)	0 (0)
RAS or BRAF unknown	4 (10.3)	0 (0)

BMI, body mass index; ECOG, Eastern Cooperative Oncology Group; MMR, mismatch repair; MSI, microsatellite instability; MSI-L, microsatellite instability low; MSS, microsatellite stable; pMMR, mismatch repair proficient

### Tolerability and recommended dose

**Figure 1:**
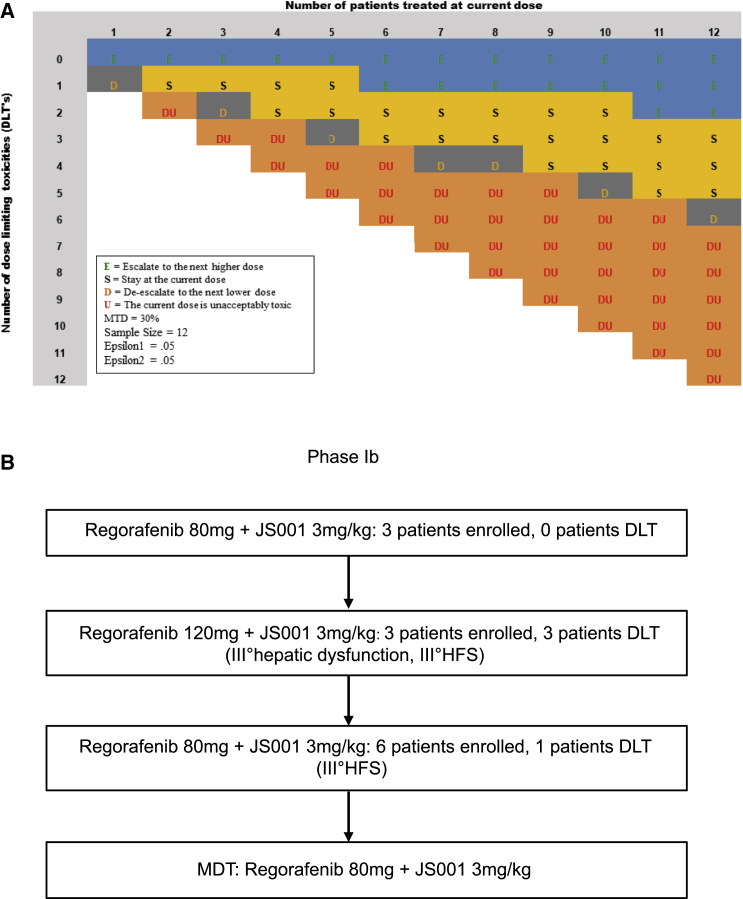
The mTPI design spreadsheet for phase Ib dose escalation (A) The spreadsheet of the modified toxicity probability interval (mTPI) method. The letters in different colors are computed based on the decision rules under the mTPI method and represent different dose-finding actions. In addition to actions de-escalate the dose (D), stay at the same dose (S), and escalate the dose (E), the table includes action unacceptable toxicity (DU), which is defined as the execution of the dose-exclusion rule in mTPI. (B) The dose escalation of phase Ib. 3 patients were enrolled at 80 mg regorafenib plus 3 mg/kg toripalimab (dose 1), and no one had dose-limiting toxicity (DLT), then 3 patients were enrolled at 120 mg regorafenib plus 3 mg/kg toripalimab (dose 2) and all patients had DLT, and then 6 patients were enrolled at dose 1 and only 1 patient had DLT (pT = 11.1%). The maximum tolerated dose (MTD) was 80 mg regorafenib plus 3 mg/kg toripalimab. HFS, hand-foot syndrome.

Twelve mCRC patients were enrolled during the dose escalation phase. Regorafenib escalated from 80 mg to 120 mg and then decreased to 80 mg according to modified toxicity probability interval (mTPI) design (Figure 1A). Three DLTs (2 grade-3 hand-food syndrome [HFS] and 1 grade-3 transaminase elevation) occurred in 3 (100%) patients in the 120 mg regorafenib cohort (Table 2). One DLT (grade 3 HFS) occurred in 9 patients in the 80 mg regorafenib cohort (Figure 1B). As the incident rate of 11.1% was less than target toxicity probability (30%), 80 mg regorafenib plus 3 mg/kg toripalimab was determined to be the maximum tolerance dose (MDT) and recommended phase II dose (RP2D) for the dose expansion of 30 patients.

**Table 2:** Treatment-related adverse events

	80 mg (n = 39)			120 mg (n = 3)		
Adverse events	<Grade 3	≥Grade 3	Total	<Grade 3	≥Grade 3	Total
Total		15 (38.5%)	37 (94.9%)		3 (100.0%)	3 (100.0%)
Hand-foot syndrome	16 (41.0%)	4 (10.3%)	20 (51.3%)	1 (33.3%)	2 (66.7%)	3 (100.0%)
Rash	10 (25.6%)	2 (5.1%)	12 (30.8%)	1 (33.3%)	0 (0.0%)	1 (33.3%)
Fever	8 (20.5%)	0 (0.0%)	8 (20.5%)	0 (0.0%)	0 (0.0%)	0 (0.0%)
Hoarseness	7 (17.9%)	0 (0.0%)	7 (17.9%)	2 (66.7%)	0 (0.0%)	2 (66.7%)
Diarrhea	6 (15.4%)	1 (2.6%)	7 (17.9%)	0 (0.0%)	0 (0.0%)	0 (0.0%)
Hypertension	5 (12.8%)	1 (2.6%)	6 (15.4%)	0 (0.0%)	2 (66.7%)	2 (66.7%)
Impaired liver function	2 (5.1%)	4 (10.3%)	6 (15.4%)	0 (0.0%)	1 (33.3%)	1 (33.3%)
Chest distress	6 (15.4%)	0 (0.0%)	6 (15.4%)	0 (0.0%)	0 (0.0%)	0 (0.0%)
Myalgia	5 (12.8%)	0 (0.0%)	5 (12.8%)	0 (0.0%)	0 (0.0%)	0 (0.0%)
Headache	3 (7.7%)	2 (5.1%)	5 (12.8%)	0 (0.0%)	0 (0.0%)	0 (0.0%)
Thrombocytopenia	3 (7.7%)	1 (2.6%)	4 (10.3%)	0 (0.0%)	0 (0.0%)	0 (0.0%)
Fatigue	4 (10.3%)	0 (0.0%)	4 (10.3%)	0 (0.0%)	0 (0.0%)	0 (0.0%)
Abdominal pain	3 (7.7%)	0 (0.0%)	3 (7.7%)	0 (0.0%)	0 (0.0%)	0 (0.0%)
Proctorrhagia	3 (7.7%)	0 (0.0%)	3 (7.7%)	0 (0.0%)	0 (0.0%)	0 (0.0%)
Bilirubin elevated	1 (2.6%)	2 (5.1%)	3 (7.7%)	0 (0.0%)	0 (0.0%)	0 (0.0%)
Leukocytosis	1 (2.6%)	0 (0.0%)	1 (2.6%)	1 (33.3%)	0 (0.0%)	1 (33.3%)
Hemoglobin reduction	2 (5.1%)	0 (0.0%)	2 (5.1%)	0 (0.0%)	0 (0.0%)	0 (0.0%)
Decreased appetite	2 (5.1%)	0 (0.0%)	2 (5.1%)	0 (0.0%)	0 (0.0%)	0 (0.0%)
Frequent premature ventricular	2 (5.1%)	0 (0.0%)	2 (5.1%)	0 (0.0%)	0 (0.0%)	0 (0.0%)
Proteinuria	1 (2.6%)	0 (0.0%)	1 (2.6%)	1 (33.3%)	0 (0.0%)	0 (0.0%)
Pruritus	1 (2.6%)	0 (0.0%)	1 (2.6%)	0 (0.0%)	1 (33.3%)	1 (33.3%)
Hypothyroidism	1 (2.6%)	0 (0.0%)	1 (2.6%)	0 (0.0%)	0 (0.0%)	0 (0.0%)
Hyperthyroidism	1 (2.6%)	0 (0.0%)	1 (2.6%)	0 (0.0%)	0 (0.0%)	0 (0.0%)
Neutropenia	0 (0.0%)	1 (2.6%)	1 (2.6%)	0 (0.0%)	0 (0.0%)	0 (0.0%)
Use of glucocorticoids			4 (10.3%)			0 (0.0%)

See also Table S1.

### Efficacy

**Figure 2:**
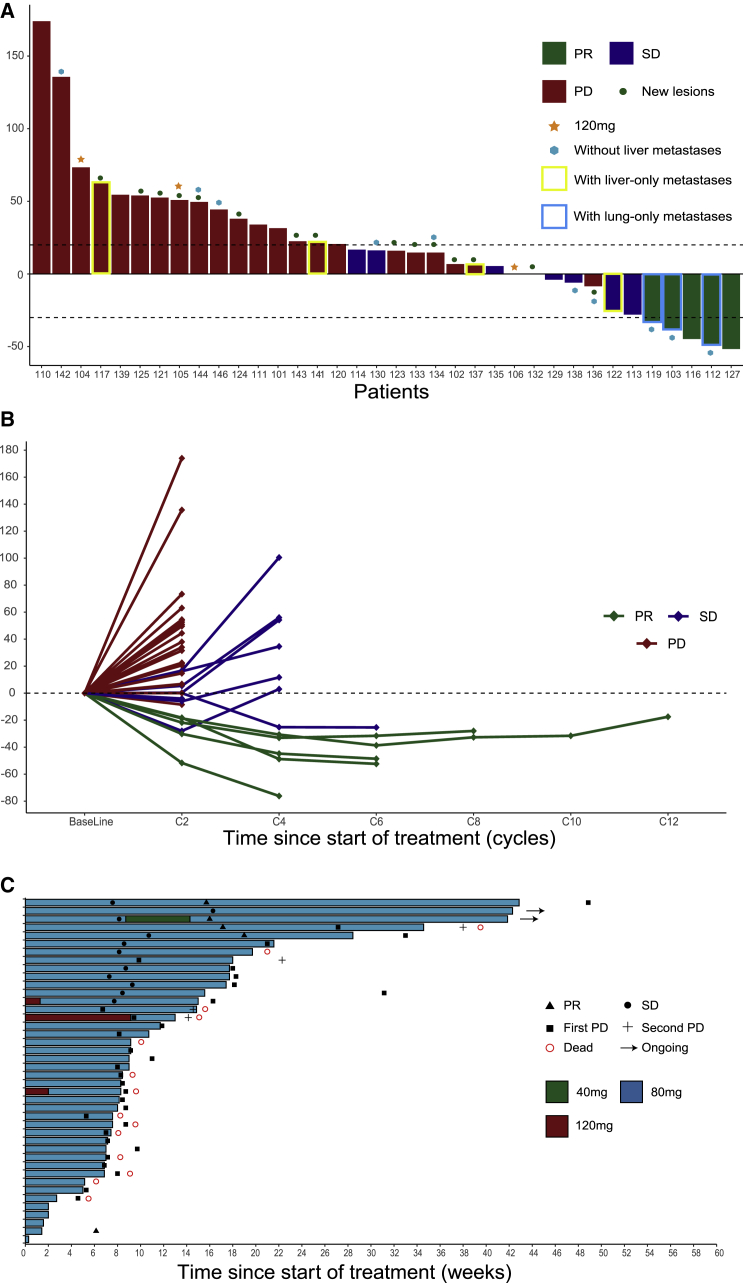
Tumor response assessment with Waterfall and Spider plots and treatment exposure and duration with Swimmer plot (A) Waterfall plot of maximum percent change in tumor size from baseline as measured according to RECIST 1.1 in 33 evaluated patients with regorafenib 80 mg and 3 evaluated patients with regorafenib 120 mg. (B) Spider plot of longitudinal change in individual tumor burden over time in RECIST percentage from baseline in 33 evaluated patients with regorafenib 80 mg and 3 evaluated patients with regorafenib 120 mg. (C) Swimmer plot according to dose level in 42 overall patients.

As of July 12, 2020, 33 patients with 80 mg regorafenib had at least one imaging tumor assessment and comprised per protocol analysis set (PPS), and 39 patients with 80 mg regorafenib comprised safety analysis set (SAF). We observed objective response in 5 patients and stable disease (SD) in 7 patients in patients with 80 mg regorafenib. The ORR was 15.2% (5/33; 95% confidence interval [CI], 5.7%–32.7%) in PPS; the disease control rate (DCR) was 36.4% (12/33; 95% CI, 21.0%–54.9%) in PPS (Figures 2A and 2B). Among 33 evaluable patients, ORR was 15.4% (2/13), 11.1% (2/18), and 50% (1/2) in *RAS* and *BRAF* wild, *RAS* mutant, and *BRAF*^*V600E*^ mutant mCRC; ORR was higher in patients without liver metastases than those with liver metastases (30% versus 8.7%); and ORR was higher in patients with lung-only metastasis (3/3; 100%) than those with liver-only metastasis (0/4; 0%; Table 3; Figure S1). Among patients with lung and liver metastases (14/33; 35.9%), ORR and DCR were 0% (0/14) and 35.7% (5/14), respectively (Table 3). Two SD patients (2/14; 14.3%) had tumor shrinkage, and one of them had obvious shrinkage in lung lesions (but stable liver lesions); one patient had disease progression after treatment (lung lesions were stable, but new lymph node lesions appeared). Tumor shrinkage of any size from baseline was observed in 9 (27.3%) patients (Figures 2A and 2B). In addition, 3 patients received 120 mg regorafenib with 1 SD and 2 progressive disease (PD) as best response (Figure 2A).

**Table 3:** Objective response rates in selected subgroups (regorafenib = 80 mg)

Subgroup	No. of patients	Objective response
All patients	33 (100%)	5 (15.2%)

**Liver metastases**		

Yes	23 (69.7%)	2 (8.7%)
No	10 (30.3%)	3 (30.0%)

**Lung metastases**		

Yes	21 (63.6%)	3 (14.3%)
No	12 (36.4%)	2 (16.7%)

**Primary site**		

Right colon	11 (33.3%)	2 (18.2%)
Left colon/rectum	22 (66.7%)	3 (13.6%)

**MSI/MMR status**		

MSS/ pMMR	32 (97.0%)	5 (15.6%)
MSI-L	1 (3.0%)	0 (0%)

***RAS/BRAF* status**		

*RAS* and *BRAF*^*V600E*^ wild type	13 (39.4%)	2 (15.4%)
*RAS* mutant	18 (54.5%)	2 (11.1%)
*BRAF*^*V600E*^ mutant	2 (6.1%)	1 (50.0%)

**Prior anti-VEGF inhibitors**		

Yes	22 (66.7%)	4 (18.2%)
No	11 (33.3%)	1 (9.1%)

**Prior anti-EGFR inhibitors**		

Yes	7 (21.2%)	2 (28.6%)
No	26 (78.8%)	3 (11.5%)

**Current treatment line**		

3	23 (69.7%)	1 (4.3%)
≥4	10 (30.3%)	4 (40.0%)

**ECOG PS score**		

0	2 (6.1%)	0 (0%)
1	31 (93.9%)	5 (16.1%)

**BMI**		

<median	16 (48.5%)	3 (18.8%)
≥median	17 (51.5%)	2 (11.8%)

**Lung-only metastases**		

Yes	3 (9.1%)	3 (100%)
No	30 (90.9%)	2 (6.7%)

**Liver-only metastases**		

Yes	4 (12.1%)	0 (0%)
No	29 (87.9%)	5 (17.2%)

See also Figure S1. ECOG PS, Eastern Cooperative Oncology Group performance status

**Figure 3:**
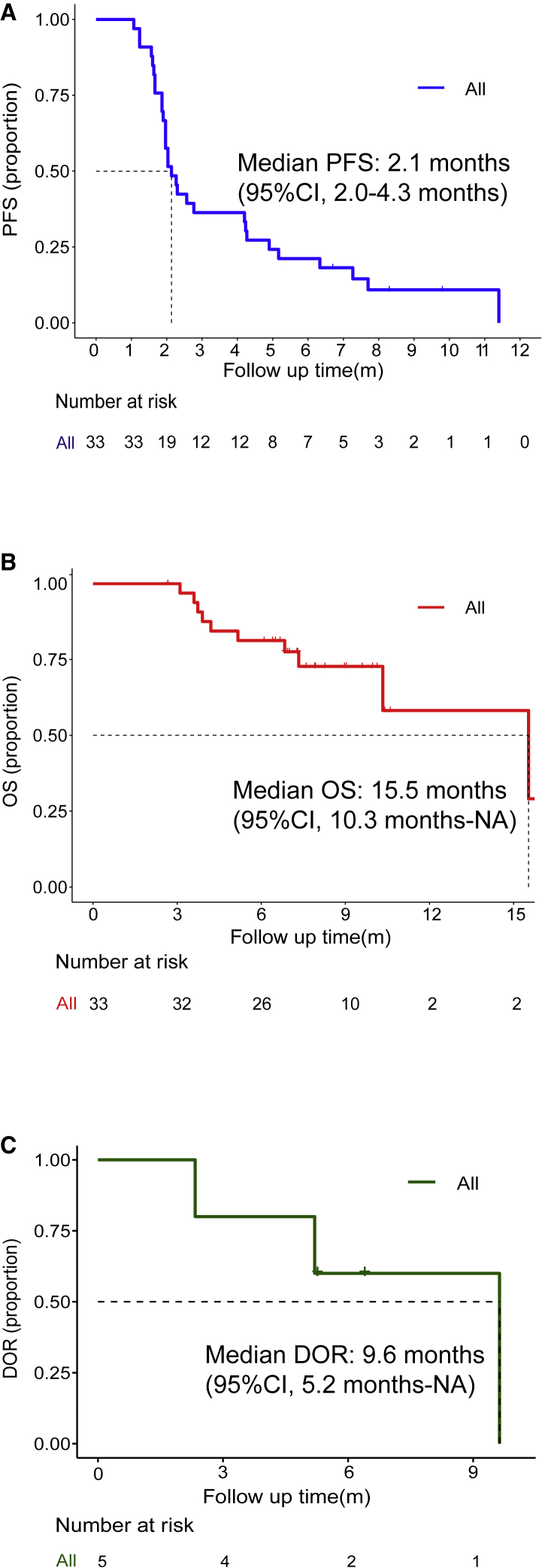
Kaplan-Meier plots of progression-free survival, overall survival, and duration of response (A and B) Kaplan-Meier plot (A) of progression-free survival (PFS) and Kaplan-Meier plot (B) of overall survival (OS) in 33 patients with regorafenib 80 mg as recommended dose. (C) Kaplan-Meier plot of duration of response (DOR) in 5 patients with partial response.

At the data cutoff of July 12, 2020, 30/33 patients had progressive disease and 23/33 patients were alive. Median progression-free survival (PFS) was 2.1 months (95% CI, 2.0–4.3 months) in PPS (Figure 3A). 6-month PFS rate was 20.5%. Median overall survival (OS) was 15.5 months (95% CI, 10.3 months-not reached [NR]; Figure 3B). 1-year OS rate was 59.8%. For the 5 patients who achieved objective response, median duration of response (DOR) was 9.6 months (95% CI, 5.2 months-NR; Figure 3C), and responses were still ongoing in 2 patients. Patients with lung-only metastasis had much longer PFS than those with liver-only metastasis (11.4 versus 2.5 months). But no differences in OS were observed between them (NR versus NR). Patients without liver metastasis also manifested with a longer median PFS of 4.1 months and median OS not reached.

### Safety

In SAF population, 94.9% patients had at least 1 treatment-related adverse event (TRAE) and 38.5% patients had at least 1 grade-3 TRAE. No grade-4 or 5 TRAEs or treatment-related deaths occurred (Table S1). Common TRAEs (≥10%) included HFS (51.3%), rash (30.8%), fever (20.5%), hoarseness (17.9%), diarrhea (17.9%), hypertension (15.4%), impaired liver function (15.4%), chest distress (15.4%), myalgia (12.8%), headache (12.8%), thrombocytopenia (10.3%), and fatigue (10.3%). The most frequent grade-3 TRAEs were HFS (10.3%) and impaired liver function (10.3%) with 2/4 of them accompanied with grade-3 hyperbilirubinemia (5.1%; Table 2). 61.5% patients experienced immune-related adverse events (irAEs), most of which were grade 1 to 2 (Table S1). Grade-3 irAE were reported in five patients (12.8%), namely rash (5.1%), impaired liver function (5.1%), and diarrhea (2.5%), which were reversible after corticosteroids treatment (Table S1). No severe adverse events occurred. In addition, 3/3 (100%) patients receiving 120 mg regorafenib developed grade-3 TRAE (Table 2).

### Discontinuation

Among the 37 patients who discontinued treatment, the most common reasons were disease progression (n = 27) followed by TRAEs (i.e., headache [n = 3], impaired liver function [n = 3], infectious pneumonia [n = 1], hand foot syndrome [n = 1], rash [n = 1], and frequent premature ventricular contractions [n = 1]; Table S1).

### Gut microbiome analysis of baseline fecal samples

**Figure 4:**
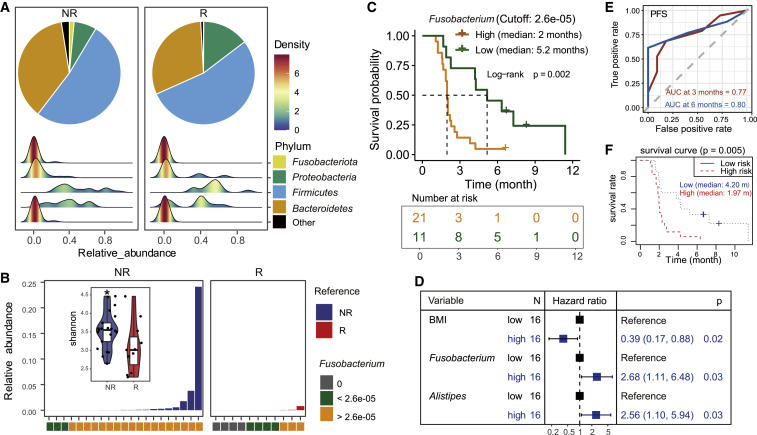
Gut microbiome analysis (A) Composition of gut microbiome at phylum level for the non-responders (NR) and responders (R), with the density plots for the distribution of the number of patients at different relative abundance region for each bacterial phylum. (B) Relative abundance of *Fusobacterium* in NR and R, with the boxplots for the alpha-diversity Shannon index of the NR and R (∗p < 0.05). (C) Kaplan-Meier plot of PFS in 32 patients with high versus low abundance of *Fusobacterium* with the best cutoff value of 2.6e−05. (D) Forest plot for multivariate Cox regression analysis of the effect of risk factors (BMI, *Fusobacterium*, and *Alistipes*) on patient’s PFS. (E) Time-dependent receiver operating characteristic (ROC) for three-variable (BMI, *Fusobacterium*, and *Alistipes*) model at PFS of 3 and 6 months. (F) PFS comparison between the high- and low-risk groups based on Cox model using Kaplan-Meier analysis. See also Tables S2–S6 and Figures S2–S4.

To explore the association of gut microbiome and the efficacy of the treatment, we performed 16S ribosomal RNA (rRNA) sequencing for the baseline fecal samples of 32 patients with the best clinical response of PR, SD, or PD. The patients were labeled with responders (R) (PR or SD; n = 11) and non-responders (NR) (PD; n = 21). Comparative analysis showed that the NR patients had remarkably increased abundance of *Fusobacteriota* and decreased *Proteobacteria* phylum (Figure 4A; Table S2). The alpha-diversity Shannon index of the baseline gut microbiome showed significant reduction in responders (Figure 4B; two-sided Wilcoxon signed-rank test; p < 0.001). Principal coordinates analysis (PCoA) of baseline bacterial operational taxonomic units (OTUs) based on the Bray-Curtis dissimilarity showed no significant difference between R and NR (Figure S2A; PERMANOVA; p = 0.822). To further identify the bacterial taxon related to the treat efficacy between R and NR patients, linear discriminant analysis (LDA)-effect size (LEfSe) analysis was employed. Twenty-three NR-enriched taxa and two R-enriched taxa were identified (Kruskal-Wallis test; LDA score ≥3; p < 0.05; Figure S3; Table S3). Among the enriched taxa, only 4 genera (*Fusobacterium*, *Alistipes*, *Bilophila*, and *Acidaminococcus*) were identified by LEfSe analysis, which were enriched in NR patients, although no enriched genus was identified in R patients (Table S3). Furthermore, using the best cutoff value of the relative abundance of *Fusobacterium* (see STAR Methods), survival analysis suggested that the patients with low abundance of *Fusobacterium* had significantly better PFS than those with high abundance of *Fusobacterium* (Figure 4C). The median PFS for patients with high-level *Fusobacterium* was much shorter than the patients with low-level *Fusobacterium* (2 versus 5.2 months; log-rank p = 0.002). Considering this observed difference of PFS might be caused by site of metastasis, we compared the clinical characteristics between high- and low-*Fusobacterium* group and found no significant difference in all the evaluated characteristics. Liver metastasis was found more frequently in high-*Fusobacterium* patients than in low-*Fusobacterium* patients, but the difference did not reach statistical significance (85.7% versus 54.5%; p = 0.088; Table S4; Figure S4). In addition, the relative abundance and positive detection rate of baseline *Fusobacterium* were higher in NR than R (Figure 4B; Table S5). Alpha or beta diversity analysis also showed no statistical differences between the patients with or without liver metastases, as well as those with lung metastases (Figures S2B–S2E).

Because the LEfSe is an algorithm for high-dimensional biomarker discovery, we combined the clinical factors of patients and the 4 genera identified by LefSe to develop a prognostic model for PFS. Univariable and multivariable CoxPH analyses resulted in a risk prediction model for PFS consisting of three covariates (body mass index [BMI], *Fusobacterium*, and *Alistipes*), which were visualized in the forest plot (Figure 4D). The baseline *Fusobacterium* and *Alistipes* levels were identified as significant risk factors of PFS with the hazard ratio (HR) of 2.68 (95% CI, 1.11–6.48; p = 0.03) and 2.56 (95% CI, 1.1–5.94; p = 0.03) respectively, while the BMI was a protective factor with HR of 0.39 (95% CI, 0.17–0.88; p = 0.02). The performance of the combination of three variates to predict PFS was illustrated by the time-dependent ROC curves (Figure 4E). The areas under the curves (AUCs) were 0.77 and 0.80 at month 3 and 6, respectively. The risk scores of patients were calculated based on the model (Table S6). The median PFSs were 1.97 months (95% CI, 1.87–2.57) and 4.2 months (95% CI, 2.04–NA) in the high-risk and the low-risk group, respectively (p = 0.005; Figure 4F).

## Discussion

Here, we report the tolerability, safety, and efficacy of regorafenib in combination with toripalimab (anti-PD-1 therapy) in MMS/pMMR/MSI-L mCRC patients as a salvage therapy.

The dose escalation in current study demonstrated the combination of 80 mg regorafenib and 3 mg/kg toripalimab had a tolerable safety profile and was selected as the RP2D. All three patients treated with 120 mg regorafenib experienced dose reduction to 80 mg, and the reduced dose was tolerable. 2.5% patients with 80 mg regorafenib experienced temporary dose reduction, and treatment delay occurred in 7.7% patients due to toxicities (Figure 2C). In comparison, the REGONIVO study also reduced the regorafenib dose from 120 to 80 mg in the dose-expansion part because of adverse events. Therefore, we consider that regorafenib 80 mg will be the recommended dose for future combination study with PD-1 antibody.

This study showed that regorafenib in combination with toripalimab has a manageable safety profile. The AEs and irAEs were generally in line with those reported for regorafenib, toripalimab, and other PD-1 or PD-L1 antibodies. No new toxicities had emerged compared with either treatment alone.^3^^,^^4^^,^^21^^,^^22^ Grade-3 irAEs were limited to 3 patients with rash or impaired liver function, and they were all manageable with corticosteroids. Moreover, no grade-4 or 5 TRAEs had occurred. The combination of 80 mg regorafenib and toripalimab seemed to have comparable safety profile with regorafenib in combinations with other PD-1/PD-L1 antibodies.^18^^,^^19^ Other anti-angiogenic molecules plus immunotherapy^14^, ^15^, ^16^ or immunotherapy plus mitogen-activated protein kinase kinase MEK inhibitor in mCRC^28^ had reported grade 3 to 4 TRAEs incidences varying from 27% to 87%. Multiple factors might contribute to the observed toxicity differences in combination studies, including different VEGFR-tyrosine kinase inhibitors (TKIs) used, ethnicity, and different proportion of regorafenib 120 mg or 160 mg adopted in other studies.

Notably, the regorafenib toripalimab combination seemed to achieve a better efficacy than regorafenib alone (ORR 1%–4%; OS 6.4–8.8 months) as salvage regimen for mCRC.^3^^,^^4^ Although there are no data for toripalimab in the field of mCRC in spite of several ongoing trials, the combination efficacy appeared better than PD-1 blockade alone and patients with MSS/pMMR mCRC are highly unlikely to respond to pembrolizumab or nivolumab.^5^ Moreover, the response was durable with a median DOR of 9.6 months reflecting the characteristics of PD-1 blockade. Thus, regorafenib in combination with toripalimab showed preliminary efficacy in unselected refractory MMS/pMMR/MSI-L mCRC patients, but the ORR was less than the statistical assumption.

The exploration of ICIs in refractory MSS/pMMR mCRC has been full of challenges. Atezolizumab combined with cobimetinib yielded a response of 3% and failed versus regorafenib.^28^ Dual checkpoint blockades of nivolumab plus ipilimumab failed with a response of 0%–10%. The combination efficacy in the current study seemed to show some advantage over the combination of regorafenib plus avelumab in REGOMUNE study with response rate of 0% and a median OS of 10.8 months^19^ and was comparable with other trials of PD-1/PD-L1 blockade combined with anti-VEGF (i.e., atezolizumab plus capecitabine and bevacizumab or pembrolizumab plus binimetinib and bevacizumab) and the retrospective study of PD-1 blockade combined with anti VEGFR (sintilimab plus fruquintinib), with ORRs ranging 8.5%–15.4%.^14^, ^15^, ^16^

As REGONIVO study reported in American Society of Clinical Oncology (ASCO), the combination of regorafenib and PD-1 antibody was widely used worldwide. However, the good response of this regimen was seldom observed in clinical practice. The current phase II study showed inferior results when compared with the cohort of mCRC patients in the phase I REGONIVO trial (ORR = 36%; median PFS 7.9 months). One possible reason is that we recruited higher proportions of patients with liver metastasis (71.4% versus 52%) and lower proportions of lymph node metastasis (45.2% versus 60%) and lung metastasis (57.1% versus 64%) than the REGONIVO study. Cumulative evidence indicated organ sites had differential impact on responses to PD-1 blockade, with lymph node, lung, and liver metastasis among the most, most/intermediate, and least responsive, respectively,^29^^,^^30^ consistent with subgroup data for liver and lung metastasis in both the current and REGONIVO studies. All patients with lymph node metastases were accompanied with other site metastases in our study that may confound their presumed response. In this trial, ORR was obviously higher in patients with lung-only metastasis than with liver-only metastasis or with both lung and liver metastases. In addition, median PFS in patients with lung-only metastasis was much longer than with liver-only metastasis. MSS mCRC patients with liver metastasis showed inferior PFS than patients without liver metastasis under PD-1 or PD-L1 checkpoint inhibition in a retrospective analysis.^31^ Besides, Yu et al.^32^ demonstrated that melanoma patients with only liver metastasis benefit less from immunotherapy than with only lung metastasis. Notably, patients without liver metastasis respond well and obviously benefit much from the combination of regorafenib plus toripalimab and deserve recommendation for this regimen. Mechanically, liver was associated with a relatively high fraction of immunosuppressive cells that is responsible for liver-metastasis-associated resistance to checkpoint blockade,^33^ and liver metastatic disease seemed to correlate with poor response to regorafenib.^34^ Besides, lung-only metastasis was associated with favorable outcomes among patients treated with regorafenib monotherapy.^34^ Patients with lung-only metastasis respond markedly to and deserve recommendation for our regimen. Another possible reason is that more patients in REGONIVO study received higher dose of regorafenib. The recommended dose of regorafenib was reduced (from 120 to 80 mg) during the dose-expansion part due to side effects. Only 60% (15/25) of patients with mCRC in REGONIVO study started with the final recommended dose of regorafenib (80 mg). However, 92.8% (39/42) of patients with mCRC initiated with 80 mg of regorafenib in our study. In addition, ECOG performance status (ECOG PS) was 0 in 7.7% of patients in our study, but in 98% in REGONIVO study, PS 0 was correlated with favorable efficacy to PD-1 blockade or regorafenib alone over PS ≥ 1.^4^^,^^34^^,^^35^ Moreover, most of the patients in this study were heavily pretreated and manifested high tumor burden, and six patients did not have computed tomography (CT) scan post-treatment due to COVID-19 pandemics. Although we only recruited two patients with *BRAF*^*V600E*^ mutations, a high response rate of 50% was observed, which was in line with genomic determinants of response to PD-1 blockade, including an enrichment of mitogen-activated protein kinase (MAPK) pathway alterations (*BRAF*) in responders^36^ and higher frequencies of tumor-associated lymphocytes associated with BRAF mutations.^37^ In conclusion, the combination of 80 mg regorafenib plus toripalimab demonstrated manageable safety profiles and showed preliminary efficacy in unselected refractory MMS/pMMR/MSI-L mCRC patients. Additional investigations of the combination in larger cohorts are warranted.

In order to identify potential biomarkers for predicting clinical response to guide patient selection and therapeutic optimization, we performed gut microbiome analysis using the pretreatment fecal samples of patients in this study. *Fusobacterium* is a genus of obligately anaerobic filamentous gram-negative rods that are members of the phylum *Fusobacteria*. Although the *Fusobacterium* species are considered opportunistic pathogens in humans and other animals, previous studies showed overabundance of *Fusobacterium* might be a risk factor for disease progression from colorectal adenoma to cancer and a therapy-predictive biomarker for colorectal cancer.^38^^,^^39^ Our results also identified the baseline *Fusobacterium* of gut microbiome as the predictive biomarker in mCRC patients under the treatment of regorafenib plus toripalimab. Because the patients with low level *Fusobacterium* in their baseline fecal samples tended to respond to the combination of regorafenib plus toripalimab, it might be a potential strategy to improve patient outcomes by reducing the abundance of the baseline *Fusobacterium*. Additionally, the development of a gut-microbiome-based prediction model for PFS highlighted the important role of gut microbiome in monitoring the clinical outcome of cancer treatment.

### Limitations of study

There are several limitations of this study. The major limitations were small sample size and patient selection (who had good PS, ECOG 0 to 1). Besides, although the efficacy analysis showed the responses of patients with lung-only metastases were much better than those with liver-only metastases, the patients’ samples of both lung-only or liver-only metastases were quite small. In addition, a similar study REGONIVO was reported previously. Although the gut microbiome testing was performed, it still lacked the analysis of dynamic changes.

## STAR★Methods

### Key resources table

**Table:** 

REAGENT or RESOURCE	SOURCE	IDENTIFIER
**Biological samples**		

Stool samples for microbiome analysis were collected from 32 patients recruited in the trial	This paper	N/A

**Chemicals and reagents**		

Regorafenib	Bayer AG	N/A
Toripalimab/JS001	Shanghai Junshi Biosciences	Table S7

**Deposited data**		

Raw data of 16S rRNA sequencing	This paper	PRJNA698295

**Software and algorithms**		

R version 3.6.1	R Project	https://www.r-project.org

### Resource availability

#### Lead contact

Further information and requests for resources and reagents should be directed to and will be fulfilled by the Lead Contact, Rui-Hua Xu (xurh@sysucc.org.cn).

#### Materials availability

This study did not generate new unique reagents.

### Experimental model and subject details

#### Ethics statement

The study, both the clinical trial and microbiome analysis, was conducted in accordance with the Declaration of Helsinki and Good Clinical Practice Guidelines after approval by the ethics board in Sun Yat-sen University Cancer Center (ID: B2019-003-05).

#### Human subjects

Chinese adults, both male and female, with histologically confirmed metastatic or unresectable MSS/MSI-L/pMMR colorectal adenocarcinoma refractory to or intolerant of fluorouracil, oxaliplatin and irinotecan based systemic treatment, were enrolled in the study. Demographic information (i.e., age and gender) was provided in Table 1, and no significant association of gender with the results of the study was found. Informed consent was obtained from all subjects.

#### Microbe strains

Baseline fecal microbiome of the enrolled patients was sequenced to detect bacterial species that existed in the feces. The bacterial phyla in the study included *Fusobacteriota, Proteobacteria, Firmicutes, Bacteroidetes,* and others. *Fusobacterium* was the species of interest in the study. Detailed species names, abundance of *Fusobacterium*, its association with clinical characteristics, and prediction for efficacy were provided in Figures 4 and S2–S4 and Tables S3–S6.

#### Other models

This study did not use any other models of animals, plants, cell lines, or primary cell cultures.

#### Sample size estimation

A total of 33 patients treated with the RP2D in the phase II dose-expansion will provide at least 90% power to show targeted efficacy of 30% ORR compared to the historical ORR of 10% using Clopper-Pearson method at a one-sided significance of 0.025, including patients with the same RP2D from phase Ib dose escalation. If > 6 patients with the RP2D have response, effectiveness could be confirmed with 90% power of test.

#### Subject allocation

The current phase Ib/II clinical trial and gut microbiome analysis was a one-arm study, with no control group, and thus all the patients were enrolled in one group and fecal samples of all the patients were collected.

### Method details

#### Study Design

The primary objective of the phase Ib dose-escalation was to evaluate tolerability and safety of toripalimab in combination with regorafenib and to determine the maximum tolerated dose (MTD) and dose limiting toxicity (DLT) of regorafenib when combined with toripalimab in patients with mCRC, providing RP2D for dose-expansion. The primary objective of the phase II dose-expansion was ORR with RP2D. Secondary objectives included safety, PFS, OS, DOR, and DCR in the patients with RP2D.

#### Patient Eligibility

The main inclusion criteria for the study were: (1) histologically confirmed metastatic and unresectable colorectal adenocarcinoma refractory to or intolerant of fluorouracil, oxaliplatin and irinotecan based systemic treatment; (2) MSS or MSI-L, or pMMR; (3) an Eastern Cooperative Oncology Group performance status (ECOG PS) of 0-1, (3) with at least 1 measurable lesion according to RECIST 1.1 criteria; (4) adequate organ function. Major exclusion criteria included: (1) previous treatment with regorafenib, PD-1/PD-L1/PD-L2 antibody or any other antibody that acts on T cell costimulatory or checkpoint pathways; (2) presence or history of autoimmune disease or status, or need of immunosuppressants; (3) human immunodeficiency virus infection, or active hepatitis, or other severe infection requiring systemic antibiotic treatment, or unexplained fever; (4) the presence of a serious comorbidity. All patients provided written informed consent for participation in the study.

#### Drug Administration and Dose Escalation Procedure

Eligible patients were orally administered with regorafenib of 80mg, 120mg, or 160 mg [po, qd (D1-D21), q4w] and intravenous toripalimab (3 mg/kg, iv, 100 mL over 1 h ± 5 min, d1 and d15, q4w) until disease progression or intolerable toxicity. In case that the lowest combination dose was intolerant, 1 mg/kg toripalimab plus 80mg regorafenib would be back up group.

DLTs were defined as any of the following toxicities occurring in the tolerability trial period (Cycle 1) determined to be related to study treatment: grade 4 neutropenia lasting for ≥ 7 days, grade ≥ 3 febrile neutropenia, grade 4 thrombocytopenia or grade ≥ 3 thrombocytopenia with hemorrhage, other hematological toxicities of grade 4 and above, grade ≥ 3 non-hematological toxicities, grade ≥ 2 neurological toxicities, and toxicities that required discontinuation of toripalimab or regorafenib ≥ 7 days.

The dose level was escalated according to a modified toxicity probability interval (mTPI) design, target level of MTD (target toxicity probability) pT = 30% and equivalence interval (EI) set between [0.25, 0.35]. The number of patients to be evaluated at each dose level was three, and then every three subjects were evaluated at the same dose level once. The maximum sample size of phase Ib trial of 12 patients. At the end of the phase Ib trial, select the dose closest to the pT as MTD and RP2D (Figure 1A).

#### Outcome Assessment

Adverse events were evaluated throughout the treatment period using the National Cancer Institute Common Terminology Criteria for Adverse Events (NCI-CTCAE V5.0). Tumor measurements were obtained using computed tomography at baseline and every 8 weeks until disease progression or at the beginning of subsequent treatment. Tumor response was evaluated per RECIST version 1.1. ORR was defined as the proportion of patients with the best overall response of complete response (CR) or partial response (PR). DCR was defined as the proportion of patients with the best overall response of CR, PR, or stable disease (SD). PFS was defined as the time from the date of enrollment until the date of disease progression or the date of death of any cause, whichever occurred first. OS was defined as the time from the date of enrollment until the date of death of any cause.

#### 16S rRNA Gene Sequencing and Data Analysis

For the collection of patient feces, samples were collected and stored at −80°C until DNA isolation. Fecal bacterial DNA was extracted using a QIAamp DNA Stool mini kit (QIAGEN) according to the manufacturer’s instructions. Bacterial DNA was extracted from fecal pellets. The V3-V4 region of the 16S rRNA encoding gene was amplified and sequenced with HiSeq Illumina platform. Raw reads were analyzed with USEARCH software (version 11) for quality control, Operational taxonomic units (OTU) clustering and taxonomy annotation. Sequences with ≥ 97% similarity were assigned to the same OTU^40^. Representative sequences for each OTU were screened for further annotation. For each representative sequence, the Silva rRNA gene database release 132 was used based on the RDP classifier algorithm.

### Quantification and statistical analysis

#### Establishment of The Prediction Model for PFS

Data analyses and representations were performed using R software v3.6.1. The optimal cut-off value for the abundance of *Fusobacterium* was determined by the surv_cutpoint () function of the “survminer” R package. Patients were classified into a high- and low- *Fusobacterium* group according to the threshold. The Kaplan–Meier survival curve combined with a log-rank test was used to compare the survival difference in the high- and low-*Fusobacterium* using the R package “survival.” Univariate and multivariate Cox Proportional Hazards Regression (CoxPH) model survival analysis were performed to identify prognostic factors (clinical factors and 4 bacterial genera). The factors were considered significant with a cut-off p ≤ 0.2. To choose the best risk prediction model for the PFS, the prognostic factors were further selected by the “step” function of R with the mode of stepwise search of “both.” The forest plot of the final CoxPH model was generated for the by the R “forestmodel” package. The predictive value of the final risk model for PFS was evaluated by time-dependent receiver operating characteristic curve (ROC) analysis using the R package “survivalROC,” and the 3- and 6-month area under the ROC Curves (AUCs) were visualized using the R packages “ggplot2.” The risk scores of patients were calculated based on risk model. The Kaplan–Meier survival curve combined with a log-rank test was further used to compare the PFS difference in the high- and low-risk patients using the R package “survival.” All the tests were two-tailed and *p* values < 0.05 were considered to be statistically significant.

### Additional resources

This study has been registered on “https://clinicaltrials.gov/,” ID: NCT03946917.

## Data Availability

Raw data of 16S rRNA gene sequencing were deposited at the NCBI database and are publicly available under the accession number listed in the Key resources table. Due to restrictions on patient privacy, the data of patients in this study is not publicly available. There was no new code developed as part of this study.

## Supplemental information

Document S1. Tables S1 and S3–S6 and Figures S1–S4

Table S2. Abundance of phyla, related to Figure 4

Table S7. Package number of toripalimab/JS001, related to STAR Methods

Document S2. Article plus supplemental information

## References

[1] Bray F., Ferlay J., Soerjomataram I., Siegel R.L., Torre L.A., Jemal A. (2018). Global cancer statistics 2018: GLOBOCAN estimates of incidence and mortality worldwide for 36 cancers in 185 countries. CA Cancer J. Clin..

[2] Wang Z.-X., Yao Y.-C., Mai Z.-J., Lin W.-H., Huang Y.-S., Jin Y., Luo H.-Y., Zhang D.-S., Wang F.-H., Wang F. (2021). Temporal change in treatment patterns of metastatic colorectal cancer and its association with patient survival: a retrospective cohort study based on an intelligent big-data platform. Engineering.

[3] Li J., Qin S., Xu R., Yau T.C., Ma B., Pan H., Xu J., Bai Y., Chi Y., Wang L., CONCUR Investigators (2015). Regorafenib plus best supportive care versus placebo plus best supportive care in Asian patients with previously treated metastatic colorectal cancer (CONCUR): a randomised, double-blind, placebo-controlled, phase 3 trial. Lancet Oncol..

[4] Grothey A., Van Cutsem E., Sobrero A., Siena S., Falcone A., Ychou M., Humblet Y., Bouché O., Mineur L., Barone C., CORRECT Study Group (2013). Regorafenib monotherapy for previously treated metastatic colorectal cancer (CORRECT): an international, multicentre, randomised, placebo-controlled, phase 3 trial. Lancet.

[5] Le D.T., Uram J.N., Wang H., Bartlett B.R., Kemberling H., Eyring A.D., Skora A.D., Luber B.S., Azad N.S., Laheru D. (2015). PD-1 blockade in tumors with mismatch-repair deficiency. N. Engl. J. Med..

[6] Overman M.J., McDermott R., Leach J.L., Lonardi S., Lenz H.J., Morse M.A., Desai J., Hill A., Axelson M., Moss R.A. (2017). Nivolumab in patients with metastatic DNA mismatch repair-deficient or microsatellite instability-high colorectal cancer (CheckMate 142): an open-label, multicentre, phase 2 study. Lancet Oncol..

[7] Wang Y., Wang M., Wu H.-X., Xu R.-H. (2021). Advancing to the era of cancer immunotherapy. Cancer Commun. (Lond.).

[8] Giannakis M., Mu X.J., Shukla S.A., Qian Z.R., Cohen O., Nishihara R., Bahl S., Cao Y., Amin-Mansour A., Yamauchi M. (2016). Genomic correlates of immune-cell infiltrates in colorectal carcinoma. Cell Rep..

[9] Topalian S.L., Hodi F.S., Brahmer J.R., Gettinger S.N., Smith D.C., McDermott D.F., Powderly J.D., Carvajal R.D., Sosman J.A., Atkins M.B. (2012). Safety, activity, and immune correlates of anti-PD-1 antibody in cancer. N. Engl. J. Med..

[10] Tada Y., Togashi Y., Kotani D., Kuwata T., Sato E., Kawazoe A., Doi T., Wada H., Nishikawa H., Shitara K. (2018). Targeting VEGFR2 with ramucirumab strongly impacts effector/ activated regulatory T cells and CD8^+^ T cells in the tumor microenvironment. J. Immunother. Cancer.

[11] Voron T., Colussi O., Marcheteau E., Pernot S., Nizard M., Pointet A.L., Latreche S., Bergaya S., Benhamouda N., Tanchot C. (2015). VEGF-A modulates expression of inhibitory checkpoints on CD8+ T cells in tumors. J. Exp. Med..

[12] Chen C.-W., Ou D.-L., Hsu C.-L., Lin L., Cheng A.-L., Hsu C. (2019). Regorafenib may enhance efficacy of anti-program cell death-1 (PD-1) therapy in hepatocellular carcinoma through modulation of macrophage polarization. J. Hepatol..

[13] Hoff S., Grünewald S., Röse L., Zopf D. (2017). Immunomodulation by regorafenib alone and in combination with anti PD1 antibody on murine models of colorectal cancer. Ann. Oncol..

[14] Mettu N.B., Twohy E., Ou F.-S., Halfdanarson T.R., Lenz H.J., Breakstone R., Boland P.M., Crysler O., Wu C., Grothey A. (2019). 533PD - BACCI: A phase II randomized, double-blind, multicenter, placebo-controlled study of capecitabine (C) bevacizumab (B) plus atezolizumab (A) or placebo (P) in refractory metastatic colorectal cancer (mCRC): An ACCRU network study. Ann. Oncol..

[15] Gou M., Yan H., E L.T., Wang Z., Si H., Chen S., Pan Y., Fan R., Qian N., Dai G. (2020). Fruquintinib combination with sintilimab in refractory metastatic colorectal cancer patients in China. J. Clin. Oncol..

[16] Lieu C.H., Davis S.L., Leong S., Leal A.D., Blatchford P.J., Sandhu G.S., Purcell W.T., Kim S.S., Van De Voorde Z., Telles R. (2020). Results from the safety lead-in for a phase II study of pembrolizumab in combination with binimetinib and bevacizumab in patients with refractory metastatic colorectal cancer (mCRC). J. Clin. Oncol..

[17] Grothey A., Tabernero J., Arnold D., De Gramont A., Ducreux M.P., O’Dwyer P.J., Van Cutsem E., Bosanac I., Srock S., Mancao C. (2018). Fluoropyrimidine (FP)+ bevacizumab (BEV) + atezolizumab vs FP/BEV in BRAFwt metastatic colorectal cancer (mCRC): Findings from Cohort 2 of MODUL–a multicentre, randomized trial of biomarker-driven maintenance treatment following first-line induction therapy. Ann. Oncol..

[18] Fukuoka S., Hara H., Takahashi N., Kojima T., Kawazoe A., Asayama M., Yoshii T., Kotani D., Tamura H., Mikamoto Y. (2020). Regorafenib plus nivolumab in patients with advanced gastric or colorectal cancer: an open-label, dose-escalation, and dose-expansion phase Ib trial (REGONIVO, EPOC1603). J. Clin. Oncol..

[19] Cousin S., Bellera C.A., Guégan J.P., Gomez-Roca C.A., Metges J.-P., Adenis A., Pernot S., Cantarel C., Kind M., Toulmonde M. (2020). REGOMUNE: a phase II study of regorafenib plus avelumab in solid tumors—results of the non-MSI-H metastatic colorectal cancer (mCRC) cohort. J. Clin. Oncol..

[20] Wang F., Wei X.-L., Feng J., Li Q., Xu N., Hu X., Liao W., Jiang Y., Lin X., Zhang Q. (2020). Clinical response and biomarker analysis of POLARIS-02 a phase II study of toripalimab, a humanized IgG4 mAb against programmed death-1 (PD-1) in patients with metastatic nasopharyngeal carcinoma. J. Clin. Oncol..

[21] Wei X.L., Ren C., Wang F.H., Zhang Y., Zhao H.Y., Zou B.Y., Wang Z.Q., Qiu M.Z., Zhang D.S., Luo H.Y. (2020). A phase I study of toripalimab, an anti-PD-1 antibody, in patients with refractory malignant solid tumors. Cancer Commun. (Lond.).

[22] Wang F., Wei X.L., Wang F.H., Xu N., Shen L., Dai G.H., Yuan X.L., Chen Y., Yang S.J., Shi J.H. (2019). Safety, efficacy and tumor mutational burden as a biomarker of overall survival benefit in chemo-refractory gastric cancer treated with toripalimab, a PD-1 antibody in phase Ib/II clinical trial NCT02915432. Ann. Oncol..

[23] Sheng X., Yan X., Chi Z., Si L., Cui C., Tang B., Li S., Mao L., Lian B., Wang X. (2019). Axitinib in combination with toripalimab, a humanized immunoglobulin G_4_ monoclonal antibody against programmed cell death-1, in patients with metastatic mucosal melanoma: an open-label phase IB trial. J. Clin. Oncol..

[24] Gopalakrishnan V., Spencer C.N., Nezi L., Reuben A., Andrews M.C., Karpinets T.V., Prieto P.A., Vicente D., Hoffman K., Wei S.C. (2018). Gut microbiome modulates response to anti-PD-1 immunotherapy in melanoma patients. Science.

[25] Routy B., Le Chatelier E., Derosa L., Duong C.P.M., Alou M.T., Daillère R., Fluckiger A., Messaoudene M., Rauber C., Roberti M.P. (2018). Gut microbiome influences efficacy of PD-1-based immunotherapy against epithelial tumors. Science.

[26] Mager L.F., Burkhard R., Pett N., Cooke N.C.A., Brown K., Ramay H., Paik S., Stagg J., Groves R.A., Gallo M. (2020). Microbiome-derived inosine modulates response to checkpoint inhibitor immunotherapy. Science.

[27] Ervin S.M., Hanley R.P., Lim L., Walton W.G., Pearce K.H., Bhatt A.P., James L.I., Redinbo M.R. (2019). Targeting regorafenib-induced toxicity through inhibition of gut microbial β-glucuronidases. ACS Chem. Biol..

[28] Eng C., Kim T.W., Bendell J., Argilés G., Tebbutt N.C., Di Bartolomeo M., Falcone A., Fakih M., Kozloff M., Segal N.H., IMblaze370 Investigators (2019). Atezolizumab with or without cobimetinib versus regorafenib in previously treated metastatic colorectal cancer (IMblaze370): a multicentre, open-label, phase 3, randomised, controlled trial. Lancet Oncol..

[29] Osorio J.C., Arbour K.C., Le D.T., Durham J.N., Plodkowski A.J., Halpenny D.F., Ginsberg M.S., Sawan P., Crompton J.G., Yu H.A. (2019). Lesion-level response dynamics to programmed cell death protein (PD-1) blockade. J. Clin. Oncol..

[30] Pires da Silva I., Lo S., Quek C., Gonzalez M., Carlino M.S., Long G.V., Menzies A.M. (2020). Site-specific response patterns, pseudoprogression, and acquired resistance in patients with melanoma treated with ipilimumab combined with anti-PD-1 therapy. Cancer.

[31] Fakih M., Sandhu J.S., Wang C., Ye J., Lee P. (2020). 495P Lack of liver metastases identifies a group of MSS metastatic colorectal cancer with potential benefit from PD-1/PD-L1 targeting. Ann. Oncol..

[32] Yu J., Green M.D., Li S., Sun Y., Journey S.N., Choi J.E., Rizvi S.M., Qin A., Waninger J.J., Lang X. (2021). Liver metastasis restrains immunotherapy efficacy via macrophage-mediated T cell elimination. Nat. Med..

[33] Lee J., Mehdizadeh S., Tsai K., Algazi A., Rosenblum M., Daud A., Bluestone J.A. (2018). Immunological insights into liver metastasis associated resistance to checkpoint blockade cancer immunotherapy. J. Immunol..

[34] Martinelli E., Sforza V., Cardone C., Capasso A., Nappi A., Martini G., Napolitano S., Rachiglio A.M., Normanno N., Cappabianca S. (2017). Clinical outcome and molecular characterisation of chemorefractory metastatic colorectal cancer patients with long-term efficacy of regorafenib treatment. ESMO Open.

[35] Mishima S., Kawazoe A., Nakamura Y., Sasaki A., Kotani D., Kuboki Y., Bando H., Kojima T., Doi T., Ohtsu A. (2019). Clinicopathological and molecular features of responders to nivolumab for patients with advanced gastric cancer. J. Immunother. Cancer.

[36] Zhao J., Chen A.X., Gartrell R.D., Silverman A.M., Aparicio L., Chu T., Bordbar D., Shan D., Samanamud J., Mahajan A. (2019). Immune and genomic correlates of response to anti-PD-1 immunotherapy in glioblastoma. Nat. Med..

[37] Bastman J.J., Serracino H.S., Zhu Y., Koenig M.R., Mateescu V., Sams S.B., Davies K.D., Raeburn C.D., McIntyre R.C., Haugen B.R., French J.D. (2016). Tumor-infiltrating T cells and the PD-1 checkpoint pathway in advanced differentiated and anaplastic thyroid cancer. J. Clin. Endocrinol. Metab..

[38] Castellarin M., Warren R.L., Freeman J.D., Dreolini L., Krzywinski M., Strauss J., Barnes R., Watson P., Allen-Vercoe E., Moore R.A., Holt R.A. (2012). Fusobacterium nucleatum infection is prevalent in human colorectal carcinoma. Genome Res..

[39] Guo S., Li L., Xu B., Li M., Zeng Q., Xiao H., Xue Y., Wu Y., Wang Y., Liu W., Zhang G. (2018). A simple and novel fecal biomarker for colorectal cancer: ratio of *Fusobacterium Nucleatum* to probiotics populations, based on their antagonistic effect. Clin. Chem..

[40] Edgar R.C. (2010). Search and clustering orders of magnitude faster than BLAST. Bioinformatics.

