# Supplementary material for: Regorafenib plus toripalimab in patients with metastatic colorectal cancer: a phase Ib/II clinical trial and gut microbiome analysis
Source: Cell Rep Med. 2021 Aug 27;2(9):100383. doi: 10.1016/j.xcrm.2021.100383 (PMC8484502; doi:10.1016/j.xcrm.2021.100383)
Supplement: Document S1. Tables S1 and S3–S6 and Figures S1–S4 [file mmc1.pdf]

Cell Reports Medicine, Volume *2*

Supplemental information

Regorafenib plus toripalimab in patients with metastatic colorectal cancer: a phase Ib/II clinical trial and gut microbiome analysis

Feng Wang, Ming-Ming He, Yi-Chen Yao, Xia Zhao, Zhi-Qiang Wang, Ying Jin, Hui-Yan Luo, Ji-Bin Li, Feng-Hua Wang, Miao-Zhen Qiu, Zhi-Da Lv, De-Shen Wang, Yu-Hong Li, Dong-Sheng Zhang, and Rui-Hua Xu

Supplementary Table S1. Summary of adverse events for RP2D (Relates to Table 2)

Patients (%)	39 (100)
Overall adverse events	37 (94.9)
Treatment-related adverse events (TRAE)	37 (94.9)
Grade 3 TRAE	15(38.5)
Grade 4-5 TRAEs or Treatment-related deaths	0 (0)
Immune-related adverse events (irAEs)	24 (61.5)
Grade 3 irAEs	5 (12.8)
Grade 4-5 irAEs	0 (0)
Severe adverse events	0 (0)
Dose reduction due to TRAE	1 (2.5)
Treatment delay due to TRAE	3 (7.7)
Discontinuation due to TRAE	10 (25.6)

Footnotes: RP2D, recommended dose; TRAE: treatment-related adverse events; irAE, immune-related adverse events

Supplementary Table S3. Results of LefSe analysis (Related to Figure 4)

Species name	group	Mean	LDA score	P value
p__Desulfobacterota.c__Desulfovibrionia.o__Desulfovibrionales	NR	3.771166	3.355917	0.02738
p__Desulfobacterota.c__Desulfovibrionia.o__Desulfovibrionales.f__Desulfovibrionaceae.g__Bilophila.s__uncultured_bacterium_g__Bilophila	NR	3.528756	3.079563	0.020388
p__Bacteroidota.c__Bacteroidia.o__Bacteroidales.f__Marinifilaceae	NR	4.058242	3.709976	0.044985
p__Firmicutes.c__Clostridia.o__Lachnospirales.f__Lachnospiraceae.g__Lachnoclostridium.s__unclassified_g__Lachnoclostridium	NR	4.020956	3.454932	0.024981
p__Bacteroidota.c__Bacteroidia.o__Bacteroidales.f__Rikenellaceae.g__Alistipes	NR	4.286034	3.88121	0.038471
p__Desulfobacterota	NR	3.771166	3.355917	0.02738
p__Fusobacteriota.c__Fusobacteriia.o__Fusobacteriales.f__Fusobacteriaceae	NR	4.175258	3.829314	0.007349
p__Firmicutes.c__Negativicutes.o__Acidaminococcales	NR	4.427439	3.982389	0.02687
p__Desulfobacterota.c__Desulfovibrionia	NR	3.771166	3.355917	0.02738
p__Fusobacteriota.c__Fusobacteriia.o__Fusobacteriales	NR	4.17551	3.829568	0.006522
p__Bacteroidota.c__Bacteroidia.o__Bacteroidales.f__Rikenellaceae	NR	4.286117	3.881285	0.034892
p__Fusobacteriota.c__Fusobacteriia.o__Fusobacteriales.f__Fusobacteriaceae.g__Fusobacterium.s__unclassified_g__Fusobacterium	NR	3.492524	3.108888	0.034073
p__Firmicutes.c__Clostridia.o__Oscillospirales.f__Oscillospiraceae	NR	4.529602	4.023316	0.049535
p__Fusobacteriota	NR	4.17551	3.829568	0.006522
p__Firmicutes.c__Negativicutes.o__Acidaminococcales.f__Acidaminococcaceae.g__Acidaminococcus.s__unclassified_g__Acidaminococcus	NR	3.800104	3.36105	0.022903
p__Fusobacteriota.c__Fusobacteriia	NR	4.17551	3.829568	0.006522

p__Desulfobacterota.c__Desulfovibrionia.o__Desulfovibrionales.f__Desulfovibrionaceae	NR	3.771166	3.355917	0.02738
p__Bacteroidota.c__Bacteroidia.o__Bacteroidales.f__Rikenellaceae.g__Alistipes.s__unclassified_g__Alistipes	NR	4.157967	3.830633	0.018925
p__Firmicutes.c__Negativicutes.o__Acidaminococcales.f__Acidaminococcaceae	NR	4.427439	3.982389	0.02687
p__Firmicutes.c__Negativicutes.o__Acidaminococcales.f__Acidaminococcaceae.g__Acidaminococcus	NR	3.922173	3.470986	0.021349
p__Fusobacteriota.c__Fusobacteriia.o__Fusobacteriales.f__Fusobacteriaceae.g__Fusobacterium	NR	4.175258	3.829314	0.007349
p__Desulfobacterota.c__Desulfovibrionia.o__Desulfovibrionales.f__Desulfovibrionaceae.g__Bilophila	NR	3.534177	3.083571	0.022659
p__Firmicutes.c__Negativicutes.o__Veillonellales_Selenomonadales.f__Veillonellaceae.g__Dialister.s__Dialister_sp__Marseille_P5638	R	4.212109	4.025834	0.039341
p__Firmicutes.c__Negativicutes.o__Veillonellales_Selenomonadales	R	5.138839	4.663844	0.022527

Footnotes: R, response (partial response or stable disease); NR, non-response (disease progression)

Supplementary Table S4. Comparison of clinical characteristics between high- and low-*Fusobacterium* group based on the baseline stool samples (Related to Figure 4)

Variable	*Fusobacterium*		*p*-value [a]
	High (n = 21)	Low (n = 11)	
Gender = Male (%)	14 (66.7)	4 (36.4)	0.142
Age >60 (%)	14 (66.7)	9 (81.8)	0.441
BMI >25 (%)	7 (33.3)	3 (27.3)	1
History = Yes (%)	8 (38.1)	2 (18.2)	0.425
Smoking = Yes (%)	4 (19.0)	1 (9.1)	0.637
Drinking = Yes (%)	3 (14.3)	2 (18.2)	1
Anti-EGFR = Yes (%)	6 (28.6)	3 (27.3)	1
Anti-VEGF = Yes (%)	12 (57.1)	7 (63.6)	1
Liver metastasis = Yes (%)	18 (85.7)	6 (54.5)	0.088
Lung metastasis = Yes (%)	10 (47.6)	8 (72.7)	0.266
Lymph metastasis = Yes (%)	8 (38.1)	6 (54.5)	0.465
Peritoneal metastasis = Yes (%)	6 (28.6)	4 (36.4)	0.703
Location = left (%)	12 (57.1)	10 (90.9)	0.106
KRAS = Yes (%)	9 (45.0)	5 (45.5)	1
RAS = Yes (%)	10 (50.0)	6 (54.5)	1

Footnotes: [a] Fisher's exact test

Supplementary Table S5. Abundacne of *Fusobacterium* (Related to Figure 4)

patientID	Response	*Fusobacterium*
Patient18	NR	0.005657264
Patient04	NR	0.000496321
Patient46	NR	2.00E-05
Patient02	NR	3.77E-05
Patient05	NR	0.002797855
Patient21	NR	0.241142461
Patient17	NR	0.003597608
Patient20	NR	0.000764259
Patient31	NR	0.002347594
Patient30	NR	9.05E-05
Patient33	NR	2.15E-05
Patient38	NR	0.000430626
Patient44	NR	1.88E-05
Patient39	NR	0.000960947
Patient41	NR	4.23E-05
Patient36	NR	0.037928427
Patient29	NR	0.000195366
Patient34	NR	0.017655703
Patient22	NR	0.000107793
Patient08	NR	4.07E-05
Patient14	NR	4.27E-05
Patient13	R	0
Patient32	R	0
Patient03	R	0
Patient06	R	0.000789133
Patient26	R	1.98E-05
Patient10	R	0.000358646
Patient11	R	2.60E-05
Patient35	R	1.98E-05
Patient24	R	0
Patient19	R	0.007977039
Patient16	R	2.14E-05

Footnotes: R, response (partial response or stable disease); NR, non-response (disease progression)

Supplementary Table S6. Risk scores of patients based on prediction model (Related to Figure 4)

patientID	PFS	PFStime	riskScore	risk
Patient13	1	6.333333333	1.565316774	high
Patient32	1	4.266666667	0.612381398	low
Patient18	1	1.866666667	1.641554452	high
Patient04	1	2.2	0.638848326	low
Patient03	1	11.4	0.609178696	low
Patient46	1	1.666666667	1.565316774	high
Patient02	1	1.566666667	0.612381398	low
Patient05	1	2.033333333	1.641554452	high
Patient06	1	3.8	0.638848326	low
Patient26	1	5.166666667	0.238322177	low
Patient10	1	4.233333333	1.641554452	high
Patient21	1	1.666666667	4.196000607	high
Patient17	1	1.966666667	1.632969262	high
Patient11	1	7.266666667	0.609178696	low
Patient35	1	4.2	0.238322177	low
Patient20	1	1.233333333	4.196000607	high
Patient24	0	6.7	0.612381398	low
Patient31	1	1.066666667	4.196000607	high
Patient30	1	1.6	0.609178696	low
Patient33	1	1.9	0.238322177	low
Patient19	0	6.633333333	0.638848326	low
Patient16	0	8.3	0.238322177	low
Patient38	1	2.3	1.632969262	high
Patient44	1	2.266666667	1.565316774	high
Patient39	1	1.966666667	1.641554452	high
Patient41	1	1.966666667	0.609178696	low
Patient36	1	2.566666667	1.641554452	high
Patient29	1	1.866666667	1.641554452	high
Patient34	1	2.766666667	1.632969262	high
Patient22	1	2.033333333	1.632969262	high
Patient08	1	2.033333333	0.609178696	low
Patient14	1	1.233333333	1.565316774	high

Footnotes: 1, patient reached disease progression; 0, patient did not reach disease progression

Figure S1

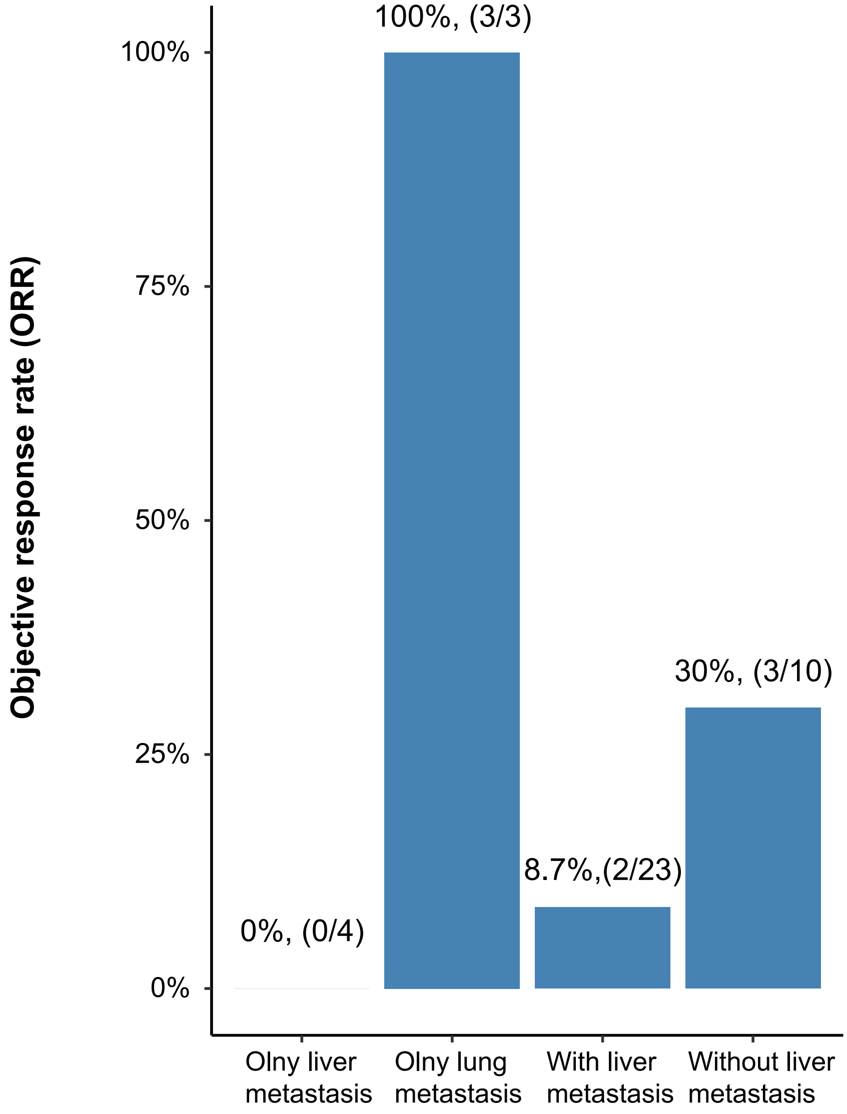

Figure S1. Objective response rate (ORR) of patients with liver metastases, without liver metastases, only with lung metastases, only with liver metastases. Related to Table 3.

Figure S2

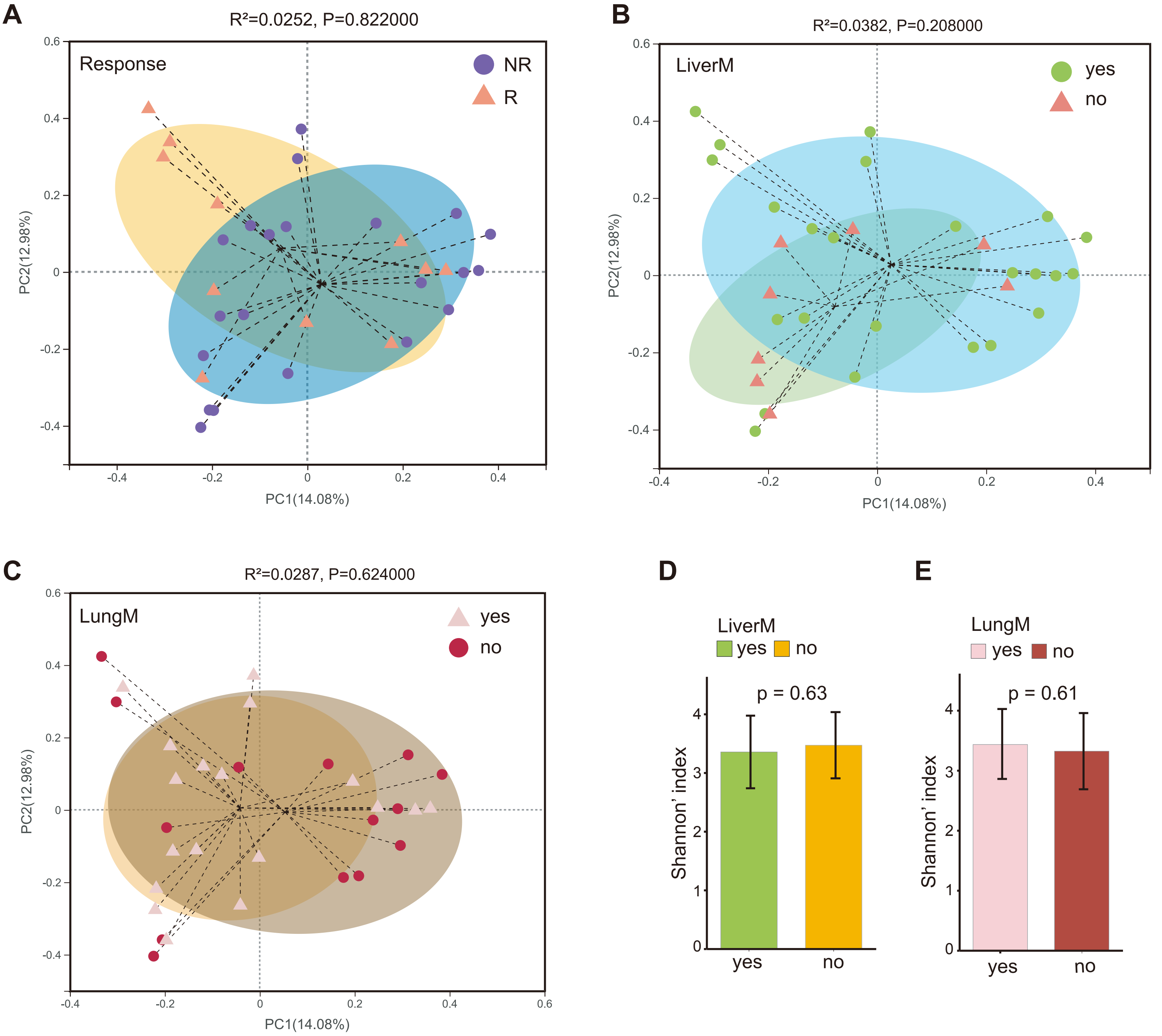

Figure S2. Alpha and beta diversity analysis for the baseline gut microbiome. Related to Figure 4. (A-C) Principal coordinates analysis (PCoA) of baseline bacterial OTUs based on the Bray-Curtis dissimilarity for the comparisions of R and NR **(A)** ,with and without metastases of liver **(B)** or lung **(C)**. **(D-E)** Comparisions of alpha diversity of baseline bacterial OTUs for the patients with and without metastases of liver **(D)** or lung **(E)**. Welch's t-test and PERMANOVA with 999 permutations were used to test the statistical differences for the alpha and beta diversity, respectively. LiverM: liver metastases; LungM: lung metastases; R, response; NR, non-response.

Figure S3

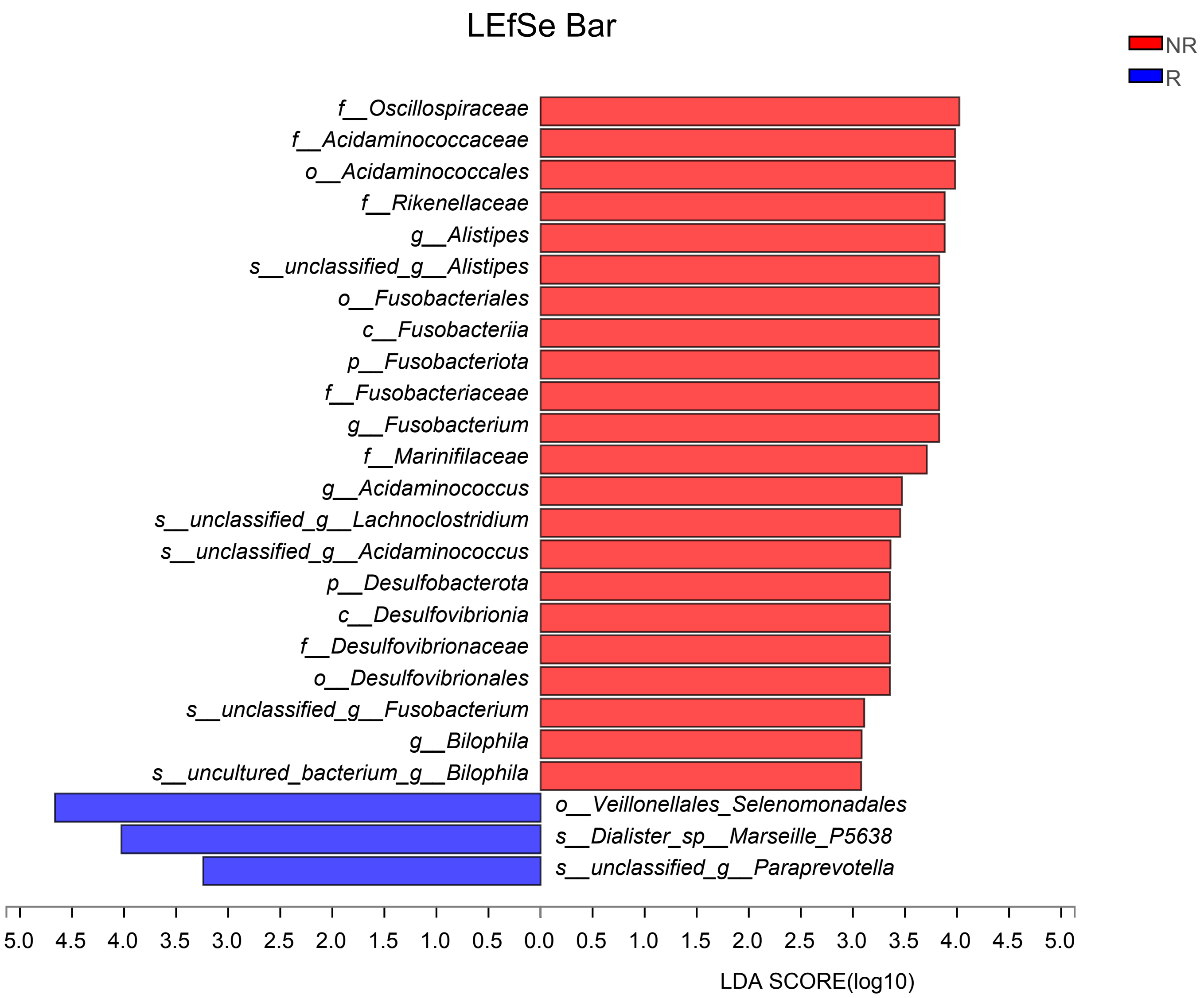

Figure S3. Linear discriminant analysis effect size (LDA) from phylum to species level shows differentially enriched taxa in NR and R groups. Related to Figure 4. R, response; NR, non-response.

Figure S4

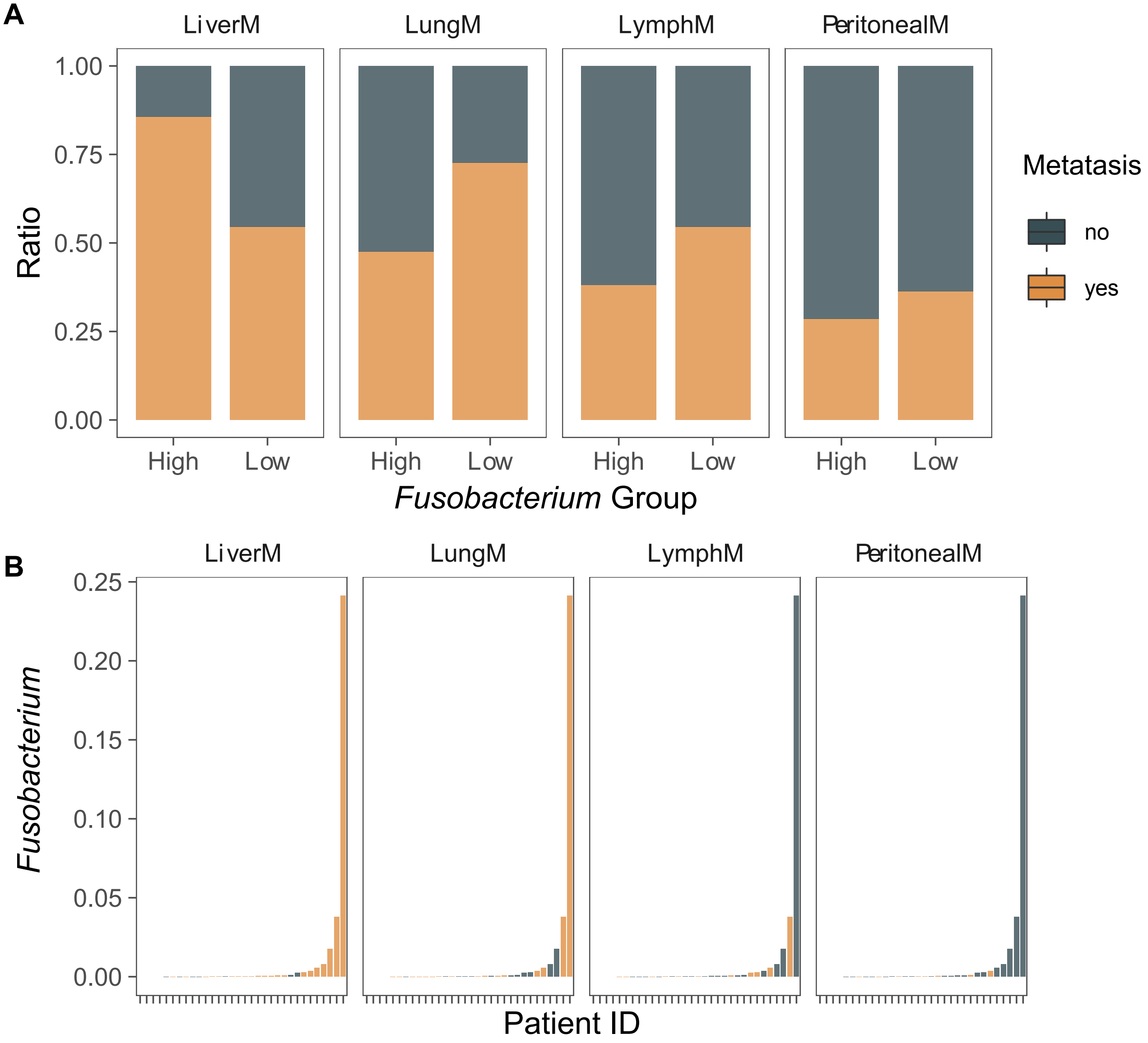

Figure S4. Comparation of the *Fusobacterium* in baseline fecal samples for different metastatic sites. Related to Table 3 and Figure 4. (A) The ratio of the patients with (yes) and without (no) metastases in high-/low- *Fusobacterium* group. **(B)** Relative abundance of *Fusobacterium* in patients with/without metastases for different metastatic sites. LiverM: liver metastases; LungM: lung metastases; LymphM: lymph node metastases; PeritonealM: peritoneal metastasis.
